# Identification of cytochrome P450 gene family and functional analysis of *HgCYP33E1* from *Heterodera glycines*

**DOI:** 10.3389/fpls.2023.1219702

**Published:** 2023-08-25

**Authors:** Jia You, Jingsheng Chen, Yanfeng Hu, Siru Wang, Jianli Wang, Tao Sun, Zhongbao Shen

**Affiliations:** ^1^ Institute of Pratacultural Science, Heilongjiang Academy of Agricultural Science, Harbin, Heilongjiang, China; ^2^ Key Laboratory of Mollisols Agroecology, Northeast Institute of Geography and Agroecology, Chinese Academy of Sciences, Harbin, Heilongjiang, China; ^3^ College of Biology and Food Engineering, Chongqing Three Gorges University, Wanzhou, China; ^4^ Chongqing Customs Technology Center, Chongqing, China

**Keywords:** soybean cyst nematode, cytochrome P450 (CYP), nematicide, abamectin, RNA interference (RNAi), detoxification

## Abstract

The cytochrome P450 (CYP) genes of nematode play a crucial role in the metabolic detoxification of xenobiotics including pesticides. *Heterodera glycines*, also known as the soybean cyst nematode, is a sedentary endoparasite that infests plant roots, causing high annual economic losses in soybean production regions globally. In this study, we identified 36 CYP genes at a genome-wide level of the *H. glycines* isolate TN10 using all CYPs from *Caenorhabditis elegans* as queries. Subsequently, a full-length cDNA of *HgCYP33E1* which was significantly up-regulated by the conventional nematicide abamectin was initially cloned from *H. glycines*. It presented significantly higher expressions in the second-stage juvenile (J2) compared to other parasitic stages of *H. glycines*. qRT-PCR analysis suggested that the expression of *HgCYP33E1* was also xenobiotically induced by soybean root exudate and the metabolites of biocontrol agents. Using RNA interference (RNAi), we investigated the function of *HgCYP33E1* in *H. glycin*e*s* parasitism and nematicide selectivity. Compared to the control and *dsGFP*-treated group, silencing of *HgCYP33E1* did not affect the J2 behaviors and the early invasion ability, while it decreased the number of J4s in soybean roots after 18-d inoculation with the *dsHgCYP33E1*-treated nematodes. In addition, knockdown of *HgCYP33E1* in *H. glycines* resulted in an increase in J2 mortality after 24-h incubation with abamectin compared to the *GFP* dsRNA-soaked and the control group. These findings revealed the potential role of *HgCYP33E1* in the xenobiotic detoxification pathway of *H. glycines*. Moreover, our data also provided valuable gene information for studying the functions of the CYP family in *H. glycines* host adaption.

## 1 Introduction

Soybean (*Glycine max* (L.) Merrill), regarded as an important oil crop which originates from China, is cultivated worldwide for feed and food products. It provide more than 59% of plant oil and 70% of vegetable protein (www.soystatus.org/). The crop is particularly critical for China’s food security, where more than 30 percent of the world’s soybean production is consumed in China, such that China has become a major importer of soybeans over the past few decades (Gale et al., 2019). Hence, to meet the increase in soybean consumption as well as to ensure food security, China has responded this demand by implementing the ‘Soybean Planting Expansion Plan’ since 2019. In 2022, more than 667,000 hectares were promoted to its soybean planting area in China. Soybean diseases can pose threats to the production because of their deleterious impacts on yield. *Heterodera glycines*, also known as soybean cyst nematode (SCN), is an obligatory endoparasite that is one of the most destructive pathogens affecting soybean production globally. In China, SCN is also considered to be a significant disease affecting soybean yield, and its presence has been reported in all soybean cultivation regions (Lian et al., 2022). SCN is particularly prevalent in two main soybean producing regions, Northeast China and the Huang-Huai Valleys in Central-East China (Chen et al., 2021; Lian et al., 2022). The annual economic losses due to SCN infestation in China were previously estimated to exceed $120 million in the infested fields (Peng et al., 2021). Given the spatial distribution of SCN in China and the impetus from China’s ‘Soybean Revitalization Plan’, this pathogen will become a more challenging threat to soybean production in China.

Currently, the measures for SCN control involves appropriate rotation system with non-host crops and cultivation of SCN-resistant cultivars (Arjoune et al., 2022). However, the genetic diversity of SCN is complex, comprising 16 virulent races. Moreover, in some soybean fields, the dominant SCN populations appear to be shifting due to the long-term planting of a single form of resistant cultivars (Lian et al., 2016; Howland et al., 2018; Chen et al., 2021). Management of SCN with host plant resistance has become complicated in recent years. The usage of nematicides has become a conventional tactic for controlling SCN globally (Gaspar et al., 2014). For example, abamectin, a highly efficient and economical nematicide, has been extensively employed to manage cyst nematodes (CNs) including SCN in certain seed treatment products and irrigation systems (Cabrera et al., 2009; Zhang et al., 2017; Clifton et al., 2018; Jensen et al., 2018a; Sasanelli et al., 2019). Therefore, it is crucial to adopt an integrated approach combining cultural practices, resistant cultivars, and nematicides to develop the most effective management strategy that reduces the losses caused by SCN in soybean fields. Furthermore, incorporating certain rotation systems that utilize susceptible varieties (with nematicides applied as seed treatments) and resistant cultivars with different R genes-based resistance can also help prevent the emergence of highly virulent populations which have overcome the SCN resistance loci of known resistant cultivars (McCarville et al., 2017; Clifton et al., 2018; Arjoune et al., 2022).

*Heterodera* species of CNs belong to soil-dwelling obligate sedentary parasites. To complete their life cycle, infective second-stage juveniles (J2s) hatching from eggs must move towards plant roots through the detection of chemical signals in soil environment, and penetrate the roots of host (Gang and Hallem, 2016). Following invasion, J2s migrate intracellularly towards the vascular cylinder to establish specialized feeding structures where they induce the formation of specialized multinucleate cells called syncytia. This sustains the subsequent sedentary life stages of CNs (Mitchum et al., 2013). As a result, throughout their life cycle, the nematodes encounter a diverse range of deleterious xenobiotic exposure both in soil environment (Lindblom and Dodd, 2006) and to plant defence chemicals from host plants (Holbein et al., 2016). Therefore, xenobiotic metabolism and extrusion functions are essential for CN survivals.

Xenobiotic detoxification has been extensively studied in the free-living nematodes and animal-parasitic nematodes (APNs) and has been confirmed to contribute significantly to the development of anthelmintics resistance in nematodes (Kotze and Prichard, 2016; Whittaker et al., 2017). Numerous researches have revealed that several enzyme families, including specific detoxification enzymes and/or drug transporters, participate in the xenobiotic detoxification pathways of nematodes. These enzymes families comprise cytochromes P450 (CYPs), UDP-glucuronosyl transferases (UGTs), glutathione S-transferases (GSTs), ATP-binding cassette transporters (ABCs), and the major facilitator superfamily (MFS) (Ghosh et al., 2012; Bygarski et al., 2014; Matoušková et al., 2016; Doyle et al., 2022). In contrast to *Caenorhabditis elegans* and APNs, the xenobiotic detoxification pathways of plant-parasitic nematodes (PPNs) to nematicides or against host defence chemicals remain largely unexplored.

The CYP superfamily has been proved to be ubiquitous across living organisms, whose importance is associated with drug resistance in mammals, insects, and nematodes (Elzaki et al., 2016; Xu et al., 2021). The genome of *C. elegans* encodes more than 80 CYP proteins (Menzel et al., 2001; Menzel et al., 2005), where several subfamilies in *C. elegans*, including CeCYP13, CeCYP14, CeCYP35, CeCYP34, CeCYP33, and CeCYP31, have been suggested to be involved in detoxification of xenobiotics (Menzel et al., 2001; Menzel et al., 2005; Laing et al., 2010; Jones et al., 2013; Jones et al., 2015; Yilmaz et al., 2019; Liu et al., 2020). In addition, most studies on CYP-mediated metabolism of xenobiotics have been conducted in APNs, such as *Haemonchus contortus* (Yilmaz et al., 2017; Kellerová et al., 2019). However, there is a serious dearth of information on the functions of CYP in parasitism and toxic metabolism of PPNs. Fragmentary evidence obtained from the pine wood nematode indicates that three *CYP* genes are related to the resistance of *Bursaphelenchus xylophilus* to nematicides such as abamectin and emamectin, as well as its pathogenicity (Xu et al., 2015; Qiu et al., 2019). Although the genomes of some important PPNs in agriculture have been sequenced, the identification of CYP gene family has only been reported in a few species, including *B. xylophilus*, *Globodera pallida*, *Meloidogyne hapla*, and *M. incognita* (Zhang et al., 2020). Therefore, the comprehension of CYPs in PPNs and their potential functions is still in the early stages.

Abamectin, which is secondary metabolite of *Streptomyces avermitis*, has been registered for the control of PPNs. It has been indicated that abamectin can induce tremor/convulsion and mydriasis on nerve/muscle cells of nematodes by releasing gamma aminobutyric acid (GABA) (Payne and Soderlund, 1993). Similar symptoms have been observed in *H. glycines* upon abamectin treatment (Jensen et al., 2018b). Additionally, our preliminary research has revealed that avermectin could be oxidized by *H. glycines* through the application of LC-MS analysis (**Figure S1**). Therefore, we postulated that an entire CYP monooxygenase system in *H. glycines* may be responsible for the detoxification of abamectin. To study the potential functions of the CYP gene superfamily in *H. glycines* parasitism and in response to xenobiotics, we conducted a genome-wide analysis of the CYP gene family in the *H. glycines* isolate TN10 genome in this study. In addition, we also analyzed the phylogenetic relationship between HgCYPs and other CYPs of *C. elegans.* A *CYP* gene, *HgCYP33E1*, which showed a significantly up-regulation upon abamectin treatment, was cloned from *H. glycines* J2s. The transcript levels of *HgCYP33E1* were evaluated in various stages and in response to soybean root exudate and the bioagent metabolic through the usage of quantitative real-time PCR (qRT-PCR). Moreover, the role of *HgCYP33E1* in *H. glycines* infection, development, reproduction, and susceptibility to abamectin was further analysed through the application of *in vitro* RNAi technique and pot assays.

## 2 Materials and methods

### 2.1 Nematode culture and plant materials

The population of *H. glycines* (race 3, HG type 0) was obtained from a naturally infested commercial soybean field in Daqing, Heilongjiang Province. The population was maintained on a susceptible soybean cultivar, ‘Hefeng25’, under greenhouse conditions at the Institute of Pratacultural Science, Heilongjiang Academy of Agricultural Science. Eggs and fresh pre-parasitic second-stage juveniles (J2s) were collected as described previously (Hu et al., 2019). Briefly, cysts were extracted from the nematode-infected soil and soybean roots using floating sieves (850-mm-pore and 250-mm-pore). Cysts were manually crushed on the surface of a 75-mm-pore sieve with a rubber stopper to release intact eggs and J2s that had hatched within cysts. Subsequently, the eggs were collected on a 25-µm pore sieve, rinsed with distilled water, and transferred to a hatching chamber at 26°C for six days for collecting hatched J2s. The seeds of soybean cv. ‘Hefeng25’ were sown in plastic pots containing a sterilized mixture of soil and sand (2:1, v/v) and grown in a greenhouse at a temperature of 21–26°C with a relative humidity at 50–65% under a 16-h light/8-h dark photoperiod.

### 2.2 Identification and phylogenetic analysis of the *CYP* genes

To identify the *CYP* genes of *H. glycines*, the amino acid sequences for all *C. elegans* CYPs listed in Wormbase (https://wormbase.org/) were utilized as queries to perform BLAST searches in the *H. glycines* isolate TN10 genome available in the WormBase ParaSite database (https://parasite.wormbase.org/Heterodera_glycines_prjna381081/Tools/Blast) with a significance cut-off of E-value < 10^−5^. The putative *HgCYP* genes were further validated through reciprocal searches of the *C. elegans* genome in the WormBase database and the non-redundant nucleotide database at the National Centre for Biotechnology Information (NCBI) BLAST server. The closest matched protein for each BLAST hit was documented as the identification of the original *H. glycines* hit. Software from Interpro (http://www.ebi.ac.uk/interpro/) was employed for functional analysis of predicted proteins. ClustalW was utilized to align the CYP proteins from *H. glycines* and *C. elegans*. Phylogenetic analyses were then performed on the comparison results, adopting the neighbor-joining method of MEGA6.0 with a Jones-Taylor/Thornton model (bootstrap test = 500 replicates).

### 2.3 Cloning *HgCYP33E1* from *H. glycines* and sequence analysis

The full-length coding sequence of *HgCYP33E1* was amplified from the cDNA of *H. glycines* J2s using specific primers (**Table S1**). PCR reactions were performed by adopting Taq PCR MasterMixII (TIANGEN, Beijing, China) with the following cycling conditions: an initial step at 94°C for 3 min, and 35 cycles of 94°C for 30 s, 59°C for 30 s, and 72°C for 96 s, followed by a final elongation time of 5 min. The PCR amplicons were purified with a PCR purification kit (TIANGEN, Beijing, China) and ligated into the pLB fast cloning vector (TIANGEN, Beijing, China) according to the manufacturer’s protocol. The resulting recombinant plasmids were transformed into *Escherichia coli* DH5α chemically competent cells (Biomed, Beijing, China) using a heat shock method. Positive clones were selected through the usage of colony PCR. Subsequently, plasmids were extracted from at least five colonies and verified via sequencing performed by COMATE biological Technology (Jilin, China). Sequence homology comparisons were conducted in Wormbase parasite database (https://parasite.wormbase.org/) and the non-redundant protein databases at the NCBI BLAST server using the BLASTX and BLASTN. Jalview Version 2.1 and ClustalW tools were employed to perform multiple sequence alignment of predicted proteins with conserved motifs across nematode species. Phylogenetic analysis of the obtained data was performed using the neighbor-joining method in MEGA6 software.

### 2.4 Xenobiotics exposure

The nematicide, abamectin (Sigma, USA), was dissolved in acetone to prepare a 10 mg/mL stock solution. To obtain the desired working concentrations (1 µg/mL, 5 µg/mL, and 10 µg/mL) for abamectin exposure of *H. glycines*, the stock solution was further diluted in distilled water. Xenobiotic treatment was carried out in a 24-cell culture plate (Corning, USA). Briefly, 500 µL of each concentration of abamectin solution was placed into a 15.6 mm diameter cell, then 20 µL nematode suspension containing approximately 1000 *H. glycines* J2s was added and gently mixed. Nematodes were incubated in the abamectin solution for 1, 2, 4, 8, 16, and 24 h in an incubator at 25°C under dark condition. The J2s exposed to a diluted acetone solution served as the control group. Then, J2s were collected and rinsed with sterile distilled water for three times, after which nematode samples were frozen in liquid nitrogen and stored at −80°C for RNA extraction.

The *Bacillus* strain *Bv-DS1* isolated previously in our laboratory (Hu et al., 2022) was used in this study. The fresh culture filtrate was prepared by growing the bacterial strain in 50 mL of LB liquid medium for 48 h at 28°C on a shaking incubator (200 rpm/min). The fermented bacteria were centrifuged at 2,500 g for 10 min, and the supernatant of the *Bv-DS1* strain was collected and filtered using a 0.22-µM Millipore filter. The prepared culture filtrate was used for nematode treatment in a 24-cell culture plate. 100 µL of undiluted culture filtrate or was added to each well, followed by the addition of a nematode suspension (approximately 1000 *H. glycines* J2s in 900 µL of water per well). The well containing 100 µL of sterilized culture broth was used as the control group. After incubation for 2, 4, 6, 8, 12, 16, and 24 h at 25°C (dark treatment), respectively; J2s were collected and washed with sterile distilled water three times. Next, nematodes were immediately frozen in liquid nitrogen and stored at −80°C until RNA extraction and gene expression analysis.

Root exudates of two soybean cultivars. ‘KangXian12’ and ‘Hefeng25’ were prepared according to the method described by Gao et al. (2018). Five seedlings for each soybean cultivar grown for four weeks were collected from the 2-L pot and gently washed with deionized water to remove vermiculites. Then, soybean seedlings were transferred to a 1-L plastic cup containing 0.5 μM CaCl_2_. In order to collect root exudates, all root systems were absolutely immerged in the solution under the dark condition for 12 h. Then, all exudate collection solutions were filtered through a 0.22-μm filter and lyophilized for subsequent treatment. A stock solution of root exudates was prepared by dissolving 10 mg of lyophilized powder in 1 mL of distilled water. Subsequently, 10 µL crude root exudate was added to each well of a 24-cell culture plate containing approximately 1000 *H. glycines* J2s in 900 µL of water per well. The plate was incubated at 25°C in the dark for 2, 4, 6, 8, 16, and 24 h, respectively. J2s exposed to sterile water were set as the control group. Nematode samples were collected and immediately frozen in liquid nitrogen for RNA extraction.

Above experiments were repeated at least three times and each treatment had at least three biological replicates. Nematode samples for RNA extraction were collected from at least three wells at each time point as a biological replicate.

### 2.5 RNA isolation and qRT-PCR analysis

Total RNA was extracted from approximately 2000 *H. glycines* nematodes in each sample using an RNAPrep Pure Micro Kit (TianGen Biotech, Beijing, China) following the manufacturer’s instructions. The quality and quantity of the extracted RNA were evaluated through a NanoDrop 2000 spectrophotometer (Thermo Fisher Scientific, Waltham, MA, USA). An equal amount (0.5 µg) of purified RNA was reverse-transcribed into cDNA using a FastKing gDNA Dispelling RT SuperMix FastKing Kit (TianGen Biotech, Beijing, China). Gene expression levels were analysed in the LightCycler® 480 real-time PCR System with AceQ qPCR SYBR Green Master Mix (Vazyme, Nanjing, China). The qRT-PCR procedure involved a preheat cycle of 95°C for 5 min, followed by 40 cycles of 10 s at 95°C and 30 s at 60°C. The primers were designed based on sequences of *HgCYP* genes retrieved from *H. glycines* database (https://scnbase.org/) using Primer 5.0 software. The sequences for the forward and reverse primers used for qRT-PCR are listed in **Supplemental Table S1**. After amplification, a melting curve analysis was performed to confirm the specificity of target genes. For each nematode sample, three biological and three technical replicates were conducted. Gene expression was normalized to the levels of a reference gene, glyceraldehyde-3-phosphate dehydrogenase (*HgGAPDH*) of *H. glycines* (Wang et al., 2014). The fold change in expression was calculated using the 2^-ΔΔCt^ method (Livak and Schmittgen, 2001).

### 2.6 *In vitro* RNA interference (RNAi) assay

The specific primers for dsRNA synthesis were designed based on the CDS sequences of *HgCYP33E1* and an unrelated *GFP* gene, which contains a T7 promoter sequence appended to the 5’ end (**Table S1**). The 787-bp fragment of *HgCYP33E1* was amplified by PCR from the recombinant pLB plasmids that contained *HgCYP33E1* inserts. Subsequently, the amplified products were used as a template for synthesizing dsRNA with a MEGAscript^®^ RNAi Kit (Thermo Fisher Scientific, Waltham, MA, USA) following the manufacturer’s protocols. The synthesized dsRNAs were examined using 1% agarose gel electrophoresis and were stored at -80°C until further use. The method of dsRNA soaking of *H. glycines* J2s was performed according to the protocol established by Urwin et al. (2002). Briefly, approximately 4,000 freshly hatched J2s were mixed with a soaking buffer containing 2 mg/mL dsRNA, 3 mM spermidine, and 50 mM octopamine, and then incubated in the dark at room temperature on a slowly shaking rotator. J2s socked in dsRNA of *GFP* gene and those soaked in buffer without dsRNA were used as control groups. After the 24-h incubation, nematodes were washed four times with the nucleoid acid-free water and collected by brief centrifugation for behavior observation, RNA extraction and qRT-PCR analysis, nematicide treatment, or infection assay. The uptake of dsRNA by *H. glycines* J2s was visualized under a fluorescence microscope (Olympus, Japan) using fluorescein isothiocyanate (FITC, 0.1 mg/ml, Sigma, USA) as a fluorescent tracer.

To examine the impact of silencing of *HgCYP33E1* on *H. glycines* infectivity and development, approximately 200 J2s were socked in *dsRNA_HgCYP33E1* solution, *dsRNA*_*GFP* solution, or buffer and subsequently inoculated on 12-day-old soybean seedlings (‘Hefeng25’) grown in pot (12-cm-diameter × 8-cm-deep) with a sterilized sand-to-soil ratio of 2:1 in a growth chamber (16 h of light and 8 h of darkness, 24°C). At two-day post-inoculation (dpi), the root systems were collected and stained with acid fuchsin solution (0.013% acid fuchsin in 0.8% acetic acid) to observe nematodes inside the roots under a stereomicroscope. After 18 dpi, the nematodes in different stages per root system were counted after acid fuchsin staining, using a microscope. The experiment was conducted three times with eight replicates for each treatment in a completely randomized experimental design.

### 2.7 Abamectin effect on viability, infectivity, and development of *H. glycines* J2s after *RNAi*

Approximately 100 *H. glycines* J2s were soaked in *dsRNA_HgCYP33E1* solution, *dsRNA*_*GFP* solution, or buffer (as control) for 24 h before being transferred into each well in a 12-well culture plate containing 1 mL of 5 µg/mL abamectin aqueous solution or 1 mL of 0.05% acetone solution (as control treatment). All plates were placed in an incubator at room temperature in the dark for 24 h. Subsequently, to determine the mortality rate of J2s, microscopic observations were conducted as previous method (Hu et al., 2019). Briefly, one drop of 1N sodium bicarbonate was added to each well. J2s that exhibited no movement during the 30-second observation period after being stimulated with sodium bicarbonate were considered dead, while any nematode that altered its body shape in response to sodium bicarbonate was classified as alive. This experiment was performed twice with six replicates for each treatment in a completely randomized experimental design.

To evaluated *H. glycines* infectivity, freshly hatched J2s were treated with *dsRNA_HgCYP33E1*, *dsRNA_GFP*, or socking buffer (as control) for 24 h before being incubated in abamectin (5 µg/mL) aqueous solutions or in acetone solution (0.05%) for 24 h at room temperature in the dark. After incubation, the nematodes were thoroughly washed with distilled water to remove abamectin. Approximately 200 J2s were inoculated on 12-day-old ‘Hefeng25’ seedlings grown in pots under the same condition as described above. At 2 dpi, each root system was collected and stained with acid fuchsin solution (0.35 acid fuchsin, 25% acetic acid) to count the number of J2s that penetrated into roots using a stereomicroscope. This experiment was conducted twice with eight replicates for each treatment in a completely randomized experimental design.

### 2.8 Data analysis

All data were subjected to one-way ANOVA with Turkey *post hoc* test (for multi-group comparisons) using GraphPad Prism version 8.0.2 software. Error bars in all figures were expressed as the mean ± the standard deviation (SD). The significance level was set at *P* < 0.05. All graphs were generated using GraphPad Prism 8

## 3 Results

### 3.1 Identification and phylogenetic analysis of the *CYP* gene family in *H. glycines*

**Figure 1:**
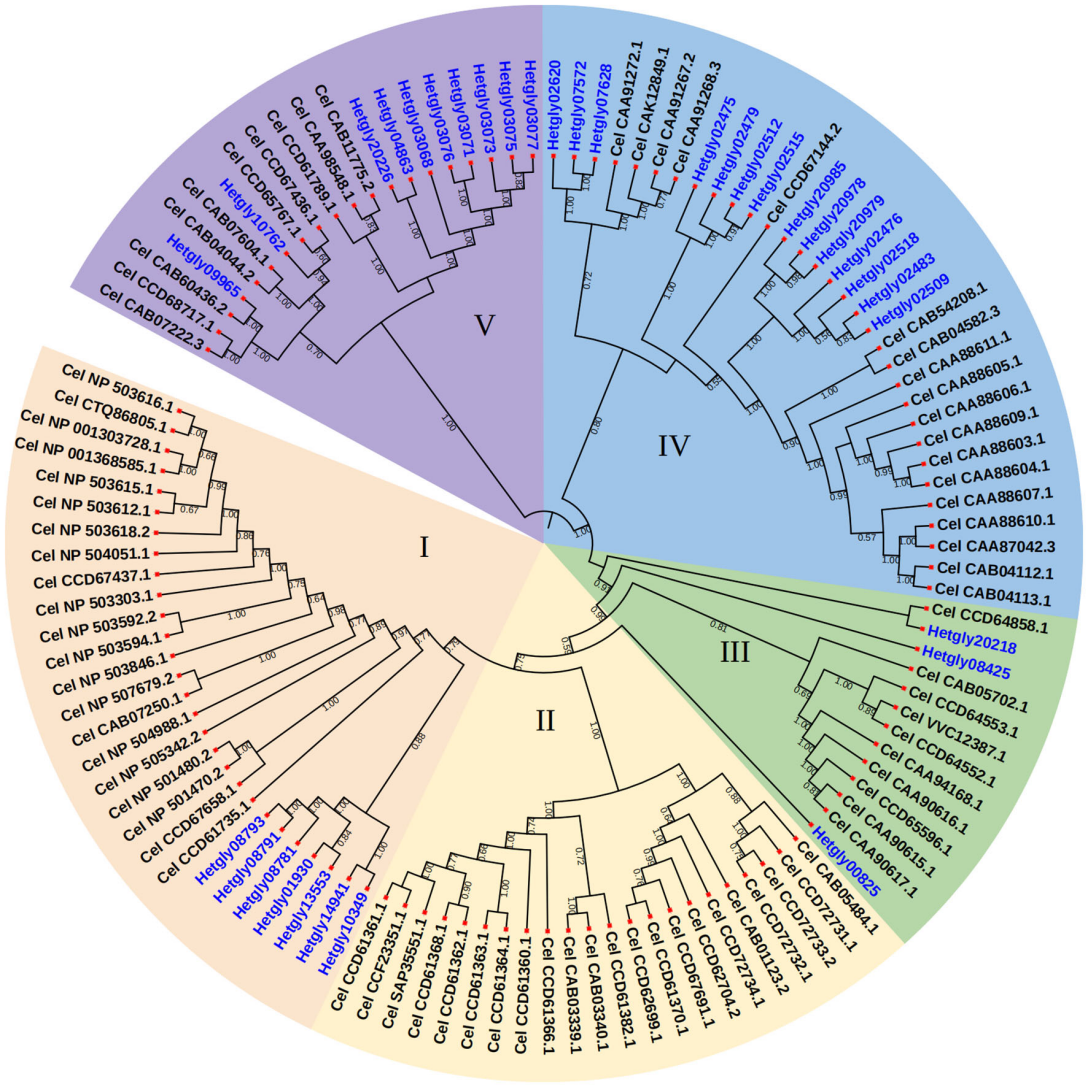
Phylogenetic relationships of CYP proteins from *H. glycines* and *C. elegans*. The protein sequences were aligned using the Clustal tool of MEGA6 software and a phylogenetic tree was constructed using MEGA6 with the neighbor-joining method. Bootstrap values were calculated from 500 replicates, and only Bootstrap values of 30% or higher were shown at each node.

The genome information of *H. glycines* (isolate TN10) (Masonbrink et al., 2019), available in the WormBase ParaSite database, was used to identify the *CYP* genes in *H. glycines*. Amino acid sequences of *CeCYP* genes from *C. elegans* in the WormBase were used as queries to screen for putative CYP proteins in *H. glycines*. Total of 36 *CYP* gene loci were identified in the 123-Mb pseudomolecule assembly of the *H. glycines* genome, responsible for encoding at least 31 full-length and 5 partial CYP proteins (**Table S2**). Among *HgCYP* gene loci, *Hetgly04863* has two transcripts. The gene IDs, gene names, and protein sequences of all identified HgCYP members are listed in **Table S2**. Our analysis revealed that *HgCYP* genes were relative evenly distributed across the eight chromosomes, with the exception of chromosome 07. The majority of *HgCYPs* (13 genes, accounting for 36.11 of the total) were clustered on chromosome 05. All identified HgCYPs possess the conserved domains of the cytochrome P450 family, including the typical heme binding loop (FxxGxxxCxG), helix-K (ExxR), and helix-C (WxxxR). After conducting phylogenetic analysis of 34 *HgCYPs* and 81 *CYPs* from *C. elegans*, a neighbor-joining phylogenetic tree was generated to visualize the results. The CYP proteins from *H. glycines* were clustered in four major sub-groups, namely I, III, IV, and V (**Figure 1**). Sub-group IV was the largest group and clustered 14 *H. glycines* CYPs together with 18 CeCYPs. Sub-group I was the second largest group, containing 7 HgCYP and 21 CeCYPs. Sub-group III consisted of 3 HgCYPs and 10 CYPs from *C. elegans*. Sub-group IV clustered 10 HgCYPs together with 10 CeCYPs. Notably, no *H. glycines* CYPs were found in sub-group II, which only contained 22 CYPs from *C. elegans*. These findings imply that specific CYP members may have evolved independently after the divergence of *H. glycines* and *C. elegans.*

To assess whether the *HgCYP* superfamily is involved in the xenobiotic detoxification of nematicides, the expression levels of individual *HgCYP* genes were examined in *H. glycines* J2s exposed to abamectin, a well-known nematicide. Note that only the expression data for the 26 *HgCYPs* are shown in **Figure S2** by evaluating the primer specificity and amplification efficiency. Due to the high degree of sequence similarity, it is difficult to design specific primers to amplify *Hetgly07572* and *Hetgly07628* separately. Therefore, the qRT-PCR results for *Hetgly07572/Hetgly07628* represent the combined expression values of these two genes and do not exhibit statistical significance relative to the control. As a result, exposure of *H. glycines* to 1 µg/mL abamectin for 2 and 8 hours generated 10 significantly (*P < 0.05, **P < 0.01) differentially expressed *HgCYPs*, with 5 up-regulated and 5 down-regulated genes (**Figure S2**). In sub-group I of *HgCYPs*, abamectin treatment resulted in an average increase in expression of *Hetgly10349* and *Hetgly13553* between 1.70- and 2.74-fold, with the exception of expression of *Hetgly08781* which was downregulated 1.95-fold at 8 h. Interestingly, *Hetgly08425* of sub-group III showed a fluctuating change in expression pattern, which was downregulated 3.98-fold after exposure 2 h and then upregulated 3.81-fold at 8 h. For all tested members of sub-group IV, abamectin treatment only caused the significant (*P* < 0.01) change in expression of *Hetgly02475*, with upregulation 3.93-fold at 8 h (**Figure S2**). Additionally, of the four differentially expressed *HgCYPs* in sub-group V, all but *Hetgly13473* was downregulated by abamectin (**Figure S2**).

### 3.2 Cloning and sequence analysis of the *HgCYP33E1* gene from *H. glycines*

**Figure 2:**
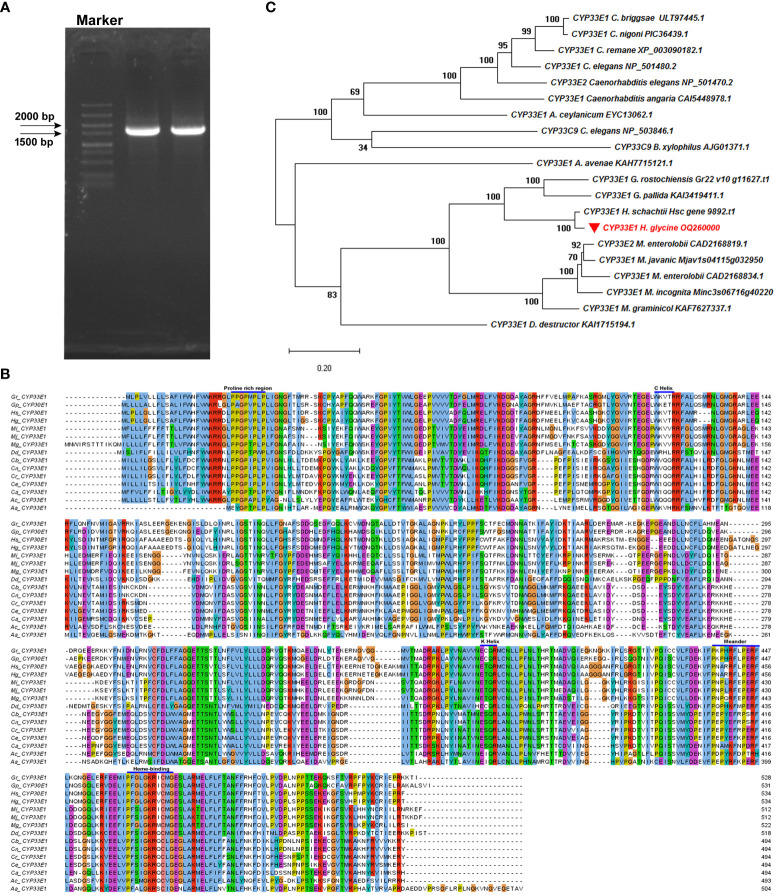
Cloning and sequencing analysis of *HgCYP33E1*. **(A)** PCR amplification cDNA of *H. glycines* J2s produced a 1602-bp band, corresponding to the full-length ORF of *HgCYP33E1*. **(B)** A multiple amino acid sequence alignment of *HgCYP33E1* with *CYP33E1* homologues from other nematodes was generated using ClustalW and Jalview tools, revealing the conserved protein domains. **(C)** Phylogenetic analysis of *HgCYP33E1* and related CYP proteins was performed using the neighbor-joining method and MEGA 6 software. Bootstrap values were calculated from 1000 replicates.

Of the five abamectin-induced *HgCYP* genes, *Hetgly10349*, *Hetgly02475*, and *Hetgly13553* were inferred to encode full-length CYP proteins. Hence, *Hetgly10349*, *Hetgly02475* and *Hetgly13553* were selected as representatives to further explore the potential function of the CYP superfamily in *H. glycines* parasitism and response to xenobiotics. Finally, only a full-length 1602-bp open reading frame (ORF) of *Hetgly13553* was successfully amplified from J2s cDNA of *H. glycines* using the gene-specific primers based on its transcript in the *H. glycines* genome (Masonbrink et al., 2019) (**Figure 2A**). The resulting ORF fragment (GenBank accession OQ260000.1) encoded a protein of 534 amino acids. High nucleotide sequence similarity was observed between the cloned ORF fragment and *Hetgly13553* (85% query cover and 99% identity) in *H. glycines* TN10 genome, as well as a transcript evm.model.chr1.1377 (100% query cover and 99% identity) in *H. glycines* X12 genome (https://scnbase.org/). We used the deduced amino acid sequence of *Hetgly13553* as a query to perform BLASTp analysis in the nonredundant protein database of NCBI and WormBase Parasite databases. It was found that *Hetgly13553* exhibited a 31.78% homology to CeCYP33E1 (NP_501480.2) of *C. elegans*, and a 35.32% identity to CYP33E1 (KAH7715121.1) of *Aphelenchus avenae*. Therefore, the CYP protein encoded by *Hetgly13553* was designated as HgCYP33E1. Furthermore, multiple homologous sequences of HgCYP33E1 were identified within phylum Nematoda, including free-living, animal-parasitic, and plant-parasitic species. Specifically, it shared 95.69%, 64.39%, and 62.71% high sequence similarity with three CYP33E1 proteins of CN species including *H. schachtii* (Hsc_gene_9892), *Globodera rostochiensis* (Gr22_v10_g11627), and *Globodera pallida* (KAI3419411.1), respectively. Additionally, HgCYP33E1 displayed a relative high sequence identity with two homologues from root-knot nematode, *Meloidogyne graminicola* (KAF7627337.1, 48.08%) and *Meloidogyne javanica* (Mjav1s04115g032950, 52.34%). A comparison of protein homology of CYP33E1 from *H. glycines* and other nematode species further revealed that HgCYP33E1 had several typical conserved domains of the cytochrome P450 family, such as heme binding loop (FxxGxxxCxG), helix-K (ExxR), and helix-C (WxxxR). Its N-terminal also contained a conserved region of proline (**Figure 2B**). A phylogenetic tree was constructed to analyze the evolutionary relationship of CYP33E1 among *H. glycines* and other species. It was observed that HgCYP33E1 was first clustered into one group with CYP33E1 proteins of PPNs. In contrast, HgCYP33E1 shared higher percentage of sequence with CYP33E1 proteins from *Globodera* and *Heterodera* nematodes, and then formed a subgroup (**Figure 2C**).

### 3.3 *HgCYP33E1* expression pattern in the different development stages of *H. glycines*

**Figure 3:**
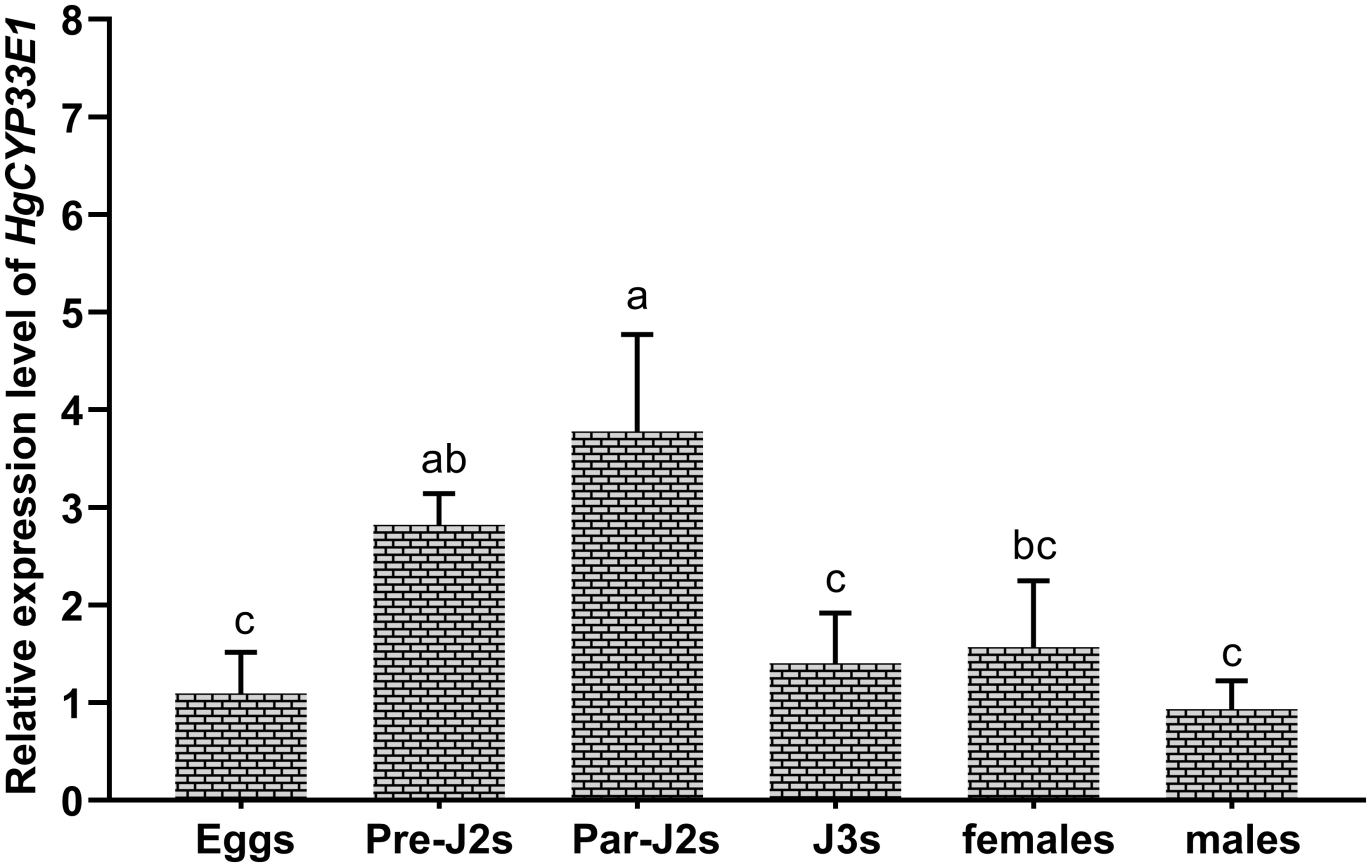
Developmental expression levels of *HgCYP33E1* in *H. glycines*. The relative transcript levels of *HgCYP33E1* were determined by qRT-PCR analysis in eggs, pre-parasitic juveniles (Pre-J2s), par-J2s, J3s, females, and males. The data are presented as mean values ± SD of three replicates. Different letters above the bars indicate statistically significant differences (*P* < 0.05) among the developmental stages, as determined by Tukey’s multiple comparison tests.

The expression levels of *HgCYP33E1* were assessed via qRT-PCR analysis of RNA samples extracted from eggs, pre-parasitic J2s, parasitic J2s, J3s, females, and males of *H. glycines*. *HgCYP33E1* was found to be expressed in all developmental stages of *H. glycines.* Notably, the pre-parasitic J2s and parasitic J2s exhibited significantly (*P* < 0.05) higher expression levels of *HgCYP33E1* compared to other parasitic stages, with transcript levels being approximately 2.58- and 3.45-fold higher than those of eggs. *HgCYP33E1* expression level was found to be similar among eggs, J3s, and females, while a lower expression was observed in the males (**Figure 3**).

### 3.4 Transcription response of *HgCYP33E1* in *H. glycines* to exposure to different doses of abamectin

**Figure 4:**
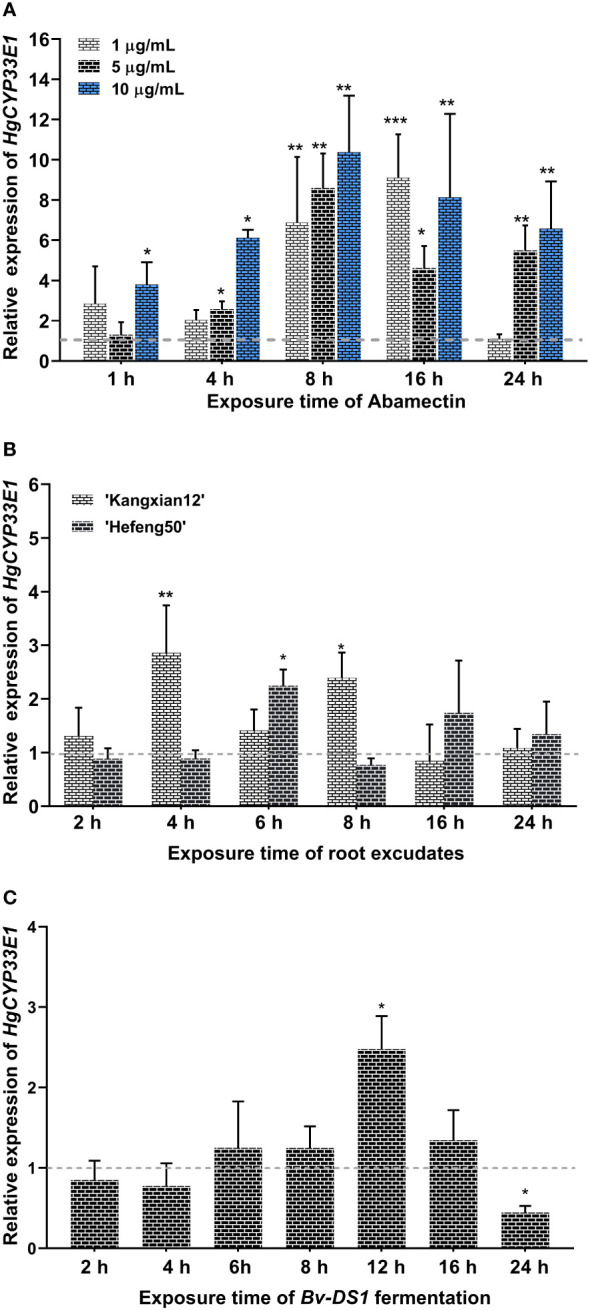
**(A)** Relative expression of *Hetgly13553* (*HgCYP33E1*) in *H. glycines* J2s treated with different concentrations of abamectin (1 µg/mL, 5 µg/mL, and 10 µg/mL) at indicated time points.The fold changes of *HgCYP33E1* expression were calculated relative to the control (J2s treated with acetone solution) using the 2^-ΔΔCt^ method. The data represent mean values of three independent experiments ± SD with three biological replicates. Asterisks above the bars indicate statistical differences between the control and treatment groups (*P < 0.05, **P < 0.01, ***P < 0.001, Student’s *t*-test). **(B)** Transcript levels of *HgCYP33E1* in *H. glycines* J2s response to 0.1 mg/L soybean root exudates from a resistant cultivar (‘Kangxian12’) and a susceptible cultivar (‘Hefeng25’). **(C)** Transcript levels of *HgCYP33E1* in response to the fermentation broth of a *Bacillus velezensis* strain YS-AT-DS1in *H. glycines* J2. qRT-PCR analysis was conducted to determine the relative transcript levels of *HgCYP33E1* at the indicated time points. The data were presented as mean values ± SD of three biological replicates. Statistical significance between the control (ddH_2_O) and treatment group was determined using Student’s *t*-test, indicated as *P < 0.05 and **P < 0.01.

Due to significantly elevated transcript levels of *HgCYP33E1* observed in abamectin-treated J2s at two time points, 2-h and 8-h (**Figure S2**), this suggests that abamectin may continuously activate *HgCYP33E1* expression in *H. glycines*. To further explore the sensitivity of *HgCYP33E1* transcript to abamectin treatment, we determined the expression levels of *HgCYP33E1* in *H. glycines* J2s exposed to different concentrations of abamectin (1 µg/mL, 5 µg/mL, and 10 µg/mL) for various time periods (1, 4, 8, 16, and 24 hours). After 1 hour exposure, both 1 µg/mL and 5 µg/mL abamectin did not cause significant changes in *HgCYP33E1* transcript levels. However, high concentrations of abamectin (10 µg/mL) significantly (*P* < 0.05) induced the expression of *HgCYP33E1* at 1 h. Significant up-regulation of *HgCYP33E1* was found at low dose of abamectin (1µg/mL) after 8 and 16 hours treatment. The transcript levels of *HgCYP33E1* increased significantly by 6.88-fold (*P* < 0.01) and 9.11-fold (*P* < 0.001), respectively, compared to nematodes treated with acetone solution (control). On the other hand, the expression levels of *HgCYP33E1* consistently increased and remained significantly up-regulation following 4, 8, 16, and 24 hours of exposure to 5 µg/mL and 10 µg/mL abamectin (**Figure 4A**). These findings indicate that the changes in expression of *HgCYP33E1* induced by abamectin are dose-dependent.

### 3.5 The expression of *Hg-CYP33E1* was xenobiotically induced by soybean root exudates and the *Bv-DS1* metabolites

Considering of the potential influence of chemicals derived from host plant, such as root exudates or defence secondary metabolites, on the behavior of the infective-juveniles of *H. glycines* in soil and the development of parasitic stages in soybean roots, it is possible that *HgCYP33E1* plays a role in the biotransformation of xenobiotics derived from host plant. To investigate this hypothesis, the relative expression levels of *HgCYP33E1* were assessed in J2s treated with root exudates from a resistant soybean cv. ‘Kangxian 12’, and a susceptible cv. ‘Hefeng25’, for different duration (2, 4, 6, 8, 16, and 24 hours). The results demonstrated that *HgCYP33E1* was significantly up-regulated after exposure to 0.1 mg/L of ‘Kangxian 12’ root exudates for 4 and 8 hours, displaying a 2.86-fold (*P* < 0.01) and 2.39-fold (*P* < 0.05) increase, respectively, compared to J2s in the control group (**Figure 4B**). In contrast, J2s exposed to root exudates of ‘Hefeng25’ for 6 hours showed significantly (*P* < 0.05) higher levels of *HgCYP33E1* transcript compared to the control worms. These results suggest that *HgCYP33E1* may be involved in responding to and processing xenobiotics present in the root exudates of soybean plants, potentially contributing to the interaction between *H. glycines* and its host plant.

Previous research suggested that certain metabolites produced by microorganisms possess nematicidal activity against *H. glycines* J2s (Lai et al., 2014). To investigate whether *HgCYP33E1* is involved in the metabolism of these xenobiotic substances from biocontrol agents, we assessed the expression levels of *HgCYP33E1* when J2s were exposed to the fermentation broth of *Bv-DS1*, known for its the biocontrol efficiency against *M. incognita* (Hu et al., 2022).The results revealed that *Bv-DS1* fermentation treatment caused a mortality rate of 76.4% in *H. glycines* J2s (**Figure S3**), suggesting that the *Bv-DS1* metabolites display toxic effects on *H. glycines* similar to those observed for *M. incognita.* J2s exposed to *Bv-DS1* fermentation did not show significant dereference in the transcript levels of *HgCYP33E1* compared to nematodes treated with the sterilized culture broth (control) at 2, 4, 6, 8, or 16 hours. However, *HgCYP33E1* expression significantly (*P* < 0.05) increased after 12 h of incubation with *Bv-DS1* fermentation. Interesting, after 24 hours of treatment, there was a remarkable reduction in *HgCYP33E1* expression (**Figure 4C**).

### 3.6 Impact of *in vitro* RNAi of *HgCYP33E1* on *H. glycines* infectivity and parasitism

**Figure 5:**
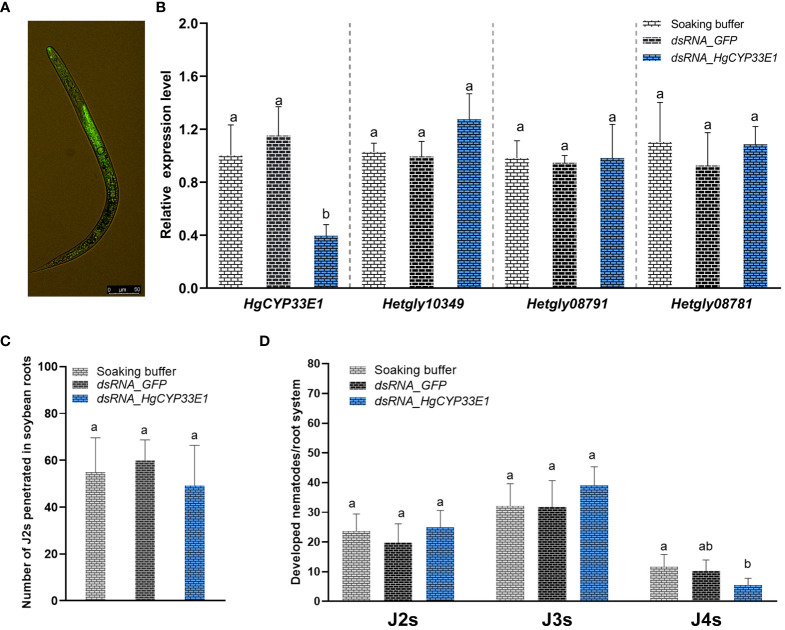
Impact of *dsRNA_HgCYP33E1* treatment on the penetration and development of *H. glycines*. **(A)** Representative fluorescent signal observed in *H. glycines* J2s soaked in dsRNA-labelled with fluorescein isothiocyanate solution, with a scale bar of 50 µm. **(B)** qRT-PCR analysis of *HgCYP* gene expression in *H. glycines* J2s after soaking in *HgCYP33E1_dsRNA* and *dsRNA_GFP* for 24 h. *H. glycines* treated in the soaking buffer without dsRNA were used as control. **(C)** The number of penetrated J2s in soybean roots at 2 days post-inoculation (dpi). **(D)** The proportion of *H. glycines* at different stages (J2, J3, and J4) in the root system at 18 dpi. The data represent mean values ± SD from three independent experiments. Statistical differences were determined using Tukey’s multiple comparison tests, with *P* < 0.05 considered significant. Different letters above the bars indicate significant differences between the groups.

To explore the role of *HgCYP33E1* in *H. glycines* parasitism, a 787-bp double-stranded RNA (dsRNA) was synthesized, containing a conservative region of *HgCYP33E1* (at 328–1160 bp). This dsRNA was then used to inhibit *HgCYP33E1* expression by soaking *H. glycines* J2s in a dsRNA solution. To ensure the specificity of silencing, the targeted fragment of *HgCYP33E1* was confirmed by BLAST search in NCBI and the *H. glycines* genome database. No significant nucleotide match for other genes was found in *H. glycines.* In addition, the sequence corresponding to targeted *dsHgCYP33E1* was further aligned with its selective three sequences (*Hetgly13554*, *Hetgly15919*, and *Hetgly20860*) with the highest scores in the results of BLAST search. No stretches of identical sequences of >6 nucleotides between the targeted *HgCYP33E1* ^328–1160^ and three no-targeted sequences were detected (**Figure S4**). Microscopic examination revealed efficient adsorption of the dsRNA labelled with fluorescein isothiocyanate, a fluorescent tracer, into J2 bodies after 24 h of soaking in the dsRNA buffer (**Figure 5A**). qRT-PCR analysis was conducted to assess the impact of RNAi on the transcript levels of *HgCYP33E1* and other *HgCYP* genes, which were clustered in sub-group I. The results showed a significant (*P* < 0.05) reduction in transcript levels of *HgCYP33E1* in nematodes that were soaked in *dsRNA_HgCYP33E1* (2 mg/mL), which was 2.5-fold and 1.9-fold lower than those in the soaking buffer without dsRNA (the control) and *dsRNA*_*GFP* solution (non-native control treatment) (**Figure 5B**). However, the expression levels of *Hetgly10349* were slightly up-regulated in *dsHgCYP33E1-*treated J2s relate to the control nematodes, but no statistical significance was found (**Figure 5B**). Subsequently, the infectivity and development of *H. glycines* on a susceptible cv. ‘Hefeng25’ was further analysed in the pot assay. The number of nematodes soaking in *dsRNA_HgCYP33E1* of buffer per root system at 2 dpi was 49.2 ± 6.8, which was slightly decreased by 10.2% when compared to the *dsRNA*_*GFP*-treated nematodes (54.8 ± 4.7), although this difference was not statistically significant (**Figure 5C**). At 18 dpi, the proportion of *H. glycines* of developmental stages in the root system was further evaluated. The roots that were inoculated with the *dsRNA_HgCYP33E1-*treated nematodes exhibited a slightly higher population of J2s and J3s, while the number of J4s was significantly (*P* < 0.05) lower, when compared to the control and *dsRNA*_*GFP*-treated group. However, there was no significant difference observed in the total number of nematodes per root system among the control, *dsRNA*_*GFP*-, or *dsRNA_HgCYP33E1-*treated nematodes (**Figure 5D**).

### 3.7 Involvement of *HgCYP33E1* in *H. glycines* susceptibility to abamectin treatment

**Figure 6:**
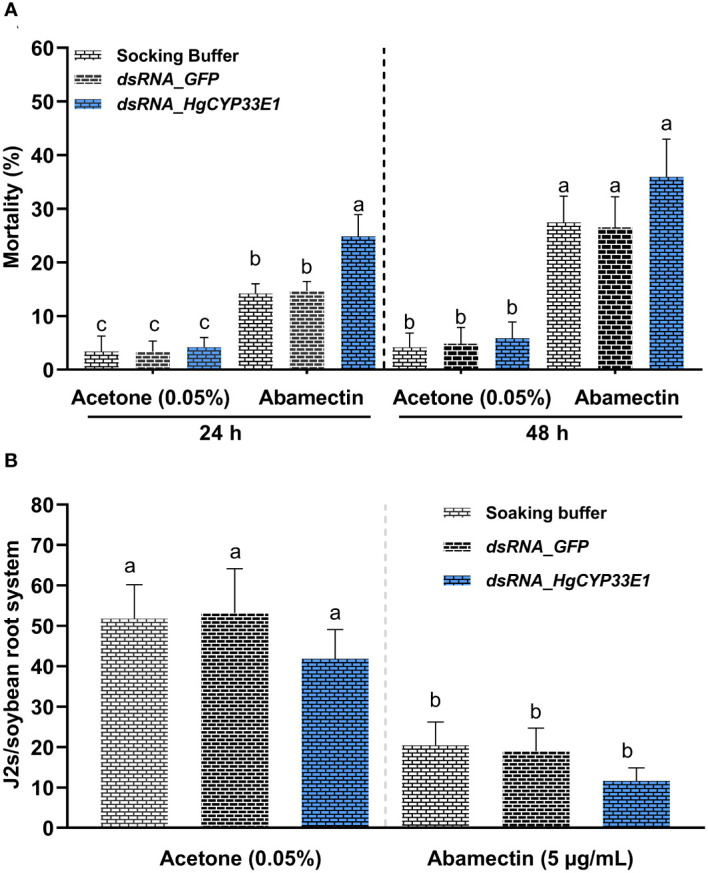
Susceptibility analysis of *H. glycines* to avermectin. **(A)** Increase in mortality of *H. glycines* with RNAi after soaking *dsRNA_HgCYP33E1* in avermectin. *H. glycines* J2s without RNAi treatment with avermectin and those soaked with acetone solution (0.05%) served as controls. The data represent mean values ± SD from two independent experiments. **(B)** Invasion rate of *H. glycines* J2s with and without dsRNA of *HgCYP33E1* after avermectin treatment. The number of J2s detected in soybean roots at 2 days after inoculation (dai) was recorded. J2s were soaked in dsRNA solution or ddH_2_O, and then were transferred to 5 µg/ml abamectin for another 24 h before the inoculation. The data are presented as means ± SD from two experiments each containing eight plants per each treatment. Statistical differences between groups were analysed using Tukey’s multiple comparison tests (*P* < 0.05) and indicated by different letters above the bars.

The analysis of gene expression suggested the induction of *HgCYP33E1* upon exposure to abamectin. Notably, a similar expression pattern of *HgCYP33E1* was observed when *H. glycines* J2s were treated with 5 µg/mL and 10 µg/mL abamectin. Consequently, we proceeded to investigate whether the knockdown of *HgCYP33E1* had any impact on *H. glycines* susceptibility to 5 µg/mL abamectin. After incubation in 0.05% acetone (v/v) solution, J2s treated with dsRNA (for both *HgCYP33E1* and *GFP)* as well as the soaking buffer control displayed similar mortality rates (**Figure 6A**). However, in contrast to J2s exposed to the buffer or *GFP*_*dsRNA*, those treated with *HgCYP33E1*_*dsRNA* showed significantly (*P* < 0.05) higher mortality rates after 24-h incubation in 5 µg/mL abamectin. The mortality rate for the *HgCYP33E1*_*dsRNA*-treated nematodes was 24.8%, whereas the *GFP*_*dsRNA*-treated and buffer-treated groups were 14.2% and 14.8%, respectively (**Figure 6A**). After 48-h abamectin treatment, the mortality rates of J2s soaked in the buffer and *GFP_dsRNA* significantly increased to 27.4% and 26.5%, respectively, compared to the control group exposed to acetone solution, but the J2 mortality for *HgCYP33E1*_*dsRNA*-treated group escalated from 5.8% to 35.9%. The findings indicate that *HgCYP33E1* may be involved in the resistance of *H. glycines* to abamectin. To further examine this hypothesis, 200 non-dsRNA or dsRNA-treated J2s, which had previously been incubated for 24 hours in 5 µg/mL of abamectin solution, were inoculated on 12-days-old ‘Hefeng25’ roots. The effect of abamectin on root invasion of *H. glycines* after *HgCYP33E1* RNAi was then assessed at 2 dpi. We observed that transient exposure to abamectin decreased *H. glycines* J2s infectivity compared to the control (0.05% acetone). The number of J2s soaked with dsRNA of *HgCYP33E1* solution per root was found to be 11.6, which was lower than the *GFP*_*dsRNA*-soaked (n = 19.0) and the buffer group (n = 20.4) following abamectin treatment (**Figure 6B**). However, statistical analysis revealed no significant difference between the control and the dsRNA of *HgCYP33E1* group.

## 4 Discussion

CYPs, which constitute the largest and most diverse superfamily of multifunctional enzymes, play important roles in the metabolism of both endogenous and exogenous compounds. The characteristics and functions of CYPs have mostly been studied in the model nematode *C. elegans* (Yilmaz et al., 2017). Recently, the availability of various PPN genomic sequences has pave the way for discovering the conserved gene families. The *CYP* gene families have been identified in four economically important PPN species including *B. xylophilus*, *G. pallida*, *M. hapla*, and *M. incognita* (Zhang et al., 2020). In this study, 36 *HgCYP* genes were initially identified based on the previous draft assembly of *H. glycines* isolate TN10 (Masonbrink et al., 2019) (**Table S2**). Undoubtedly, the identified *HgCYP* gene family provides candidate genes for further functional characterization of *CYPs* in *H. glycines* parasitism. Notably, the number of *HgCYPs* superfamily in the *H. glycines* genome was found to be considerably smaller than that of the free-living nematodes *C. elegans* (81 *CYPs*) (Menzel et al., 2001) and *C. briggsae* (74 *CYPs*) (Zhang et al., 2020). However, the total gene number of *HgCYPs* was similar to that of two PPN species, *G. pallida* with 40 *CYPs* and *M. incognita* with 38 *CYPs*. These observations suggest that the CYP family in PPNs may lack the extensive gene duplication events relative to free-living nematodes, potentially due to their endoparasitic lifestyles and narrow host range. Interestingly, there are notable discrepancies in the CYP family among the three *B. xylophilus* isolates from different regions, ranging from 44 members in CN-Bx1 to 56 members in JP-Ka4C1 (Zhang et al., 2020). This variability may reflect the adaptive evolution of the CYP family expansion in different isolates, which can aid *B. xylophilushave* in surviving and adapting different host environments. Therefore, it would be intriguing to explore whether this family undergo expansion in the genomes of different avirulent and virulent *H. glycines* isolates through comparative analysis. Such research would enhance our comprehension of the role of the CYP family in the virulence evolution of *H. glycines*. Phylogenetic analysis revealed that CYPs from *H. glycines* and *C. elegans* were classified into five sub-groups, with most *H. glycines* CYPs clustered in group I, IV, and V, whereas group II consisted only of *C. elegans* CYPs (**Figure 1**). This observation suggests that this gene family undergoes rapid evolution and species-specific expansion in nematodes. In addition, the putative function of these CYPs may be linked to the differential feeding habits of *H. glycines* and *C. elegans.*

In *C. elegans*, the xenobiotic detoxification pathway has two successive phases. The principal phase 1 enzymes in this pathway contain CYPs, which can hydroxylate xenobiotics (Lindblom and Dodd, 2006). When nematodes are exposed to xenobiotics, the transcription levels of *CYP* genes are rapidly induced to initiate the detoxification processes. For instance, exposure of *C. elegans* to various xenobiotics, including pesticides, leads to a significant increase in the expression levels of some *CYP* gene subfamilies such as *CeCYP13*, *CeCYP14*, *CeCYP35*, *CeCYP34*, *CeCYP33*, and *CeCYP31* (Menzel et al., 2001; Menzel et al., 2005; Jones et al., 2013; Yilmaz et al., 2019; Liu et al., 2020). In *H. contortus*, the induction of *CYP1*, *CYP3*, and *CYP5* transcript levels by ivermectin has been reported (Kellerová et al., 2019). Additionally, the enhanced expression levels of a *CYP* gene belonging to the *CYP34/35* subfamily has been associated with benzimidazoles resistance in *H. contortus* (Yilmaz et al., 2017). Although nematicides with lower toxicity to humans and the environment, such as abamectin, are widely used for controlling PPNs in agriculture, there have been no reports showing the involvement of *CYPs* in sensitivity of PPNs to commonly-used nematicides. Recently, a new class of bioactivated nematicides, selectivins, has been identified, displaying promise in selectively controlling a wide variety of PPNs (Burns et al., 2023). Using a yeast-based system expressing 19 *CYPs* from *M. incognita*, it was confirmed that CYP4731A3 is mainly responsible for the bioactivation of selectivins in *M. incognita* (Burns et al., 2023). In present study, evidence was provided that avermectin-treated J2s of *H. glycines* contained obvious hydroxy-avermectin products (**Figure S1**). This finding suggests that *H. glycines* might be capable of metabolizing avermectin through the detoxification enzymatic system. To identify the putative *CYP* genes related to abamectin detoxification in *H. glycines*, we used qRT-PCR to examine the expression pattern of the *HgCYP* family in *H. glycines* following exposure to 1 µg/mL abamectin, which was administered at an effective dose, known to be toxic to PPNs (Zhang et al., 2017; Sasanelli et al., 2019). The expression levels of 5 *HgCYPs* were significantly up-regulated by abamectin (**Figure S2**). By searching the GenBank database, we found that the abamectin-induced *HgCYPs* belonged to the *CYP13* and *CYP33* gene subfamilies. Previous studies have found that several *CYP* isoforms belonging to these families are associated with the detoxification of xenobiotics in nematodes (Menzel et al., 2001; Kulas et al., 2008; Xu et al., 2015; Liu et al., 2020). Recently, Wram et al. (2022) used RNA-seq to investigate the transcript response of *M. incognita* to four new nematicides (fluazaindolizine, fluopyram, oxamyl, and fluensulfone). They found that these nematicides induced considerable transcript changes in xenobiotic detoxification system, including 56 up-regulated *CYP* genes. This study, along with our findings, suggest that the elevated transcript level of specific *CYP* genes may be involved in the tolerance of PPNs to nematicides. However, further research is necessary to determine the exact role of these upregulated *CYPs* in nematicides detoxification. Additionally, in this study, we observed a differential regulation response among four sub-groups of *HgCYPs* in *H. glycines* after exposure to abamectin. Specially, only *Hetgly02475* from sub-group IV, which contains 14 *HgCYPs*, was significantly induced by abamectin (**Figure S2**). Similarly, the expression profiles of *CYPs* are highly diverse in J2s of *M. incognita* exposed to fluazaindolizine, fluopyram, oxamyl, or fluensulfone (Wram et al., 2022). Upregulated *CYP* genes were primarily detected when *M. incognita* J2s were treated with fluazaindolizine and fluensulfone. Moreover, only two *CYP* members, *Minc3s00305g09802* and *Minc3s00532g13848*, were differently expressed across all four nematicides (Wram et al., 2022). This revealed that specific CYP genes or sub-families in different nematode species may be activated by different nematicides.

To gain more information about the *HgCYPs* involved in nematicide detoxification, three abamectin-induced *HgCYPs* (*Hetgly10349*, *Hetgly02475*, and *Hetgly13553*) with full-length ORFs were pre-selected for functional analysis. Among these, *HgCYP33E1* was successfully clone and used for further study on its roles in *H. glycines* parasitism and resistance to nematicide (**Figure 2A**). Sequence alignment demonstrated that HgCYP33E1 shares high identity with HgCYP33E2 (Hetgly01930) and other CYP33E1 proteins of PPNs (**Figure 2B**). This level of homology in the primary protein structure may also support a similarity of biological function. In *M. incognita*, the expression of *minc3s06716g40220*, responsible for encoding a homologue of HgCYP33E1, was significantly induced by the nematicide fluensulfone (Wram et al., 2022). Accordingly, the present study provides compelling evidence that exposure to abamectin significantly increased the expression of *HgCYP33E1* in *H. glycines.* Furthermore, there was an enhanced response in *HgCYP33E1* expression with increasing doses of abamectin (**Figure 4A**). Taken together, these findings imply that CYP33E1 proteins represent the major isoforms of the CYP33 subfamily and may be associated with nematicide metabolism in PPNs.

It has been reported that the expression pattern of parasitic genes in PPNs can provide insight into their biological and physiological functions (Mitchum et al., 2013). In this study, high expression levels of *HgCYP33E1* was detected in both pre- and post-parasitic J2 stages (**Figure 3**), which are critical stage for *H. glycines* in terms of host finding and early infection. This finding suggests that HgCYP33E1 may play a crucial role in *H. glycines’* response to xenobiotics present in the soil environment and host root exudates. Plant root exudates contain a variety of compounds, such as phenolic, flavonoids, and terpenes, which can regulate the attraction, repulsion, and feeding behaviors of PPNs. Some secondary metabolites in plants have been shown to contribute to nematode resistance during interactions between PPNs species and their hosts (Lin et al., 2017; Gao et al., 2018; Torto et al., 2018; Ansari et al., 2020). Additionally, certain benefit microbiomes in the rhizospheric soil can produce multitude of volatile organic compounds that exhibit strong nematicidal activity (Seong et al., 2021). It has been reported that these xenobiotic metabolites can induce the expression of the detoxification genes, including the *CYP* genes, in PPNs. For example, in *B. xylophilushave*, *CYP29A3* was upregulated by L(-)-carvone, a plant metabolite (Chen et al., 2022). Zhang et al. (2020) reported that the gene expansion of *CYP* family is linked to the detoxification of terpenes, which are main defence chemicals in pine wood against parasites. As expected, the transcript levels of *HgCYP33E1* peaked when *H. glycines* was exposed to root exudates from a resistance soybean for 4 hours and a susceptible cultivar for 6 hours, respectively (**Figure 4B**). Moreover, our results demonstrate that the transcript levels of *HgCYP33E1* were also significantly induced when J2s were incubated with *Bv-DS1* fermentation for 12 hours (**Figure 4C**), suggesting CYP33E1 might be a multifunctional CYP450 enzyme in *H. glycines* with multiple substrates and roles in xenobiotic detoxification. However, *HgCYP33E1* then significantly decreased after 24 hours of the fermentation treatment, which could be attributed to an increase in J2 mortality resulting in the reduction in transcript levels of *HgCYP33E1* (**Figure S3**).

*In vitro* RNAi technique has been utilized to investigate gene functions in PPNs, including *H. glycines* (Banerjee et al., 2017). In *H. glycines* J2s treated with *dsRNA_HgCYP33E1*, a strongly reduced transcript levels of *HgCYP33E1* was detected, while expression levels of its gene homologous in same *HgCYP* subfamily remained unaffected (**Figure 5**). This indicates that RNAi-mediated suppression of *HgCYP33E1* was efficient and highly target-specific. In *C. elegans*, knockdown of *CYP33E1* by RNAi has been shown to positively affect the defecation phenotype (Liu et al., 2012). Another study reported that silencing of *CeCYP33E1 via* RNAi caused a partial decrease in 2,2’5,5’-tetrachlorobiphenyl hydroxylation (Schäfer et al., 2009). However, inhibition of *HgCYP33E1* expression did not result in pronounced abnormalities in J2 behaviors or a significant decrease in early invasion ability compared to control nematodes (**Figure 5C**). Possible explanations for this finding are that the CYP33 subfamily may have redundant function, and other CYP members with similar functions could compensate for the loss of *HgCYP33E1*. HgCYP33E1 showed almost the same degree of amino acid sequence homology to CeCYP33E1 (31.78%) and CeCYP33C9 (31.22%), which both belong to the xenobiotic-inducible CYP33 subfamily (Menzel et al., 2001; Kulas et al., 2008). A homolog of *CeCYP33C9* in *B. xylophilus*, *BxCYP33C9*, has been shown to be involved in nematode pathogenicity (Xu et al., 2015). Additionally, we observed that silencing of *HgCYP33E1* interfered with the later developmental stages of *H. glycines* at 18 dpi (**Figure 5D**), implying that *CYP33E1* in *H. glycines* may have a similar function to *CYP33C9* in *B. xylophilus* in detoxifying defence chemicals from host plants. Furthermore, we found that RNAi of *HgCYP33E1* increased the sensitivity of *H. glycines* to abamectin after 24 hours of exposure (**Figure 6A**), suggesting that *HgCYP33E1* might be associated with the biotransformation of abamectin. Loss of other *CYP33* genes in nematodes has been shown to lead to the similar phenotype, with a significant increase in susceptibility to pesticides. For example, knock down of *CYP33E2* resulted in decrease in *C. elegans* resistance to eicosapentaenoic acid (Kulas et al., 2008). Similarly, silencing of *BxCYP33C9*, *BxCYP33C4*, and *BxCYP33D3* in *B. xylophilus* have been reported to increase mortality when nematodes exposed to two commonly used nematicides, abamectin and emamectin (Xu et al., 2015). These findings, together with our results, indicate that the putative function in CYP33-mediated metabolism of xenobiotics is highly conserved in the phylum Nematoda and may also play a role in pesticide detoxification in PPNs. Interestingly, even though incubating the *HgCYP33E1*_*dsRNA-*treated nematodes in abamectin for 48 hours resulted in higher mortality rates compared to the control worms, there was no statistical significant difference between the control and the dsRNA of *HgCYP33E1* (**Figure 6A**). This could potentially be due to other related *HgCYP* genes being induced by abamectin, which may compensate for the knocking-down of *HgCYP33E1*. Another possible reason for this is that the effect of dsRNA on *HgCYP33E1* expression is transient. Previous studies has also mentioned the short durability of RNAi effects in PPNs (Roderick et al., 2012; Kumari et al., 2017). Thus, it is not surprising that we did not observe a significant difference in the number of J2s in soybean roots between the control and the dsRNA of *HgCYP33E1* group after abamectin treatment (**Figure 6B**). On the other hand, it is well known that abamectin is a potent neurotoxin that impairs movement or paralyzes nematodes (Payne and Soderlund, 1993; Jensen et al., 2018b). Although the control group had a lower mortality rate compared to the ds*HgCYP33E1*-treated worms, the most of the control nematodes lost their ability to move and infect due to the neurotoxicity of abamectin.

In conclusion, our study has presented important gene information for studying the role of the CYP family in *H. glycines*. Our data also demonstrated that *HgCYP33E1* is a xenobiotic-inducible *CYP* gene by pesticides and metabolites from host plants and biocontrol microorganisms. Our finding highlight that interfering with the expression of *HgCYP33E1* can affect the development and sensitivity of *H. glycines* to abamectin, suggesting that *HgCYP33E1* plays a role in the biotransformation of xenobiotic substances. However, further research is needed to explore the specific functions of *HgCYP33E1* in metabolizing pesticides or metabolites.

## Data availability statement

The datasets presented in this study can be found in online repositories. The names of the repository/repositories and accession number(s) can be found in the article/**Supplementary Material**.

## Author contributions

YH conceived and designed the experiments. JY, JC, YH, SW, JW, and TS performed the experiments. JY, JC, and YH analyzed the data. JY, JC, and YH wrote the original manuscript. YH and ZS reviewed and edited the manuscript. All authors read and approved the final manuscript.
