# Supplementary material for: Identification of cytochrome P450 gene family and functional analysis of HgCYP33E1 from Heterodera glycines
Source: Front Plant Sci. 2023 Aug 25;14:1219702. doi: 10.3389/fpls.2023.1219702 (PMC10485556; doi:10.3389/fpls.2023.1219702)
Supplement: Supplementary file 1 [file DataSheet_1.docx]

Supplementary Material

# Supplementary Figures and Tables

## Supplementary Figures


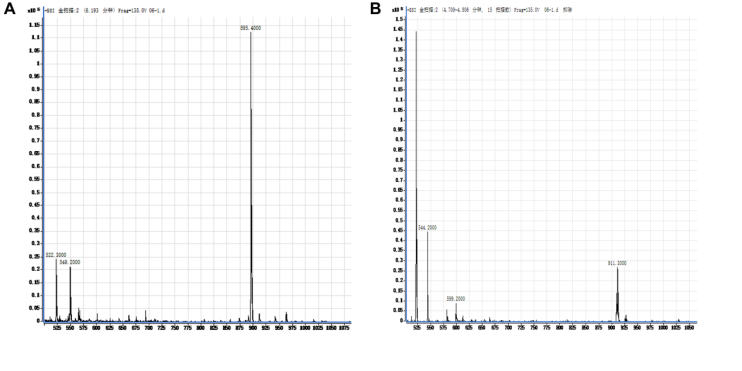


**Supplementary Figure 1.** Abamectin metabolism in *H. glycines* J2s. (A) The negative control. The representative LC-MS showed the metabolism of abamectin without incubation with *H. glycines.* (B) LC-MS analyses showed that abamectin could be oxidized by *H. glycines.* The *x*-axis represents retention time and *y*-axis represents signal abundance of compounds.


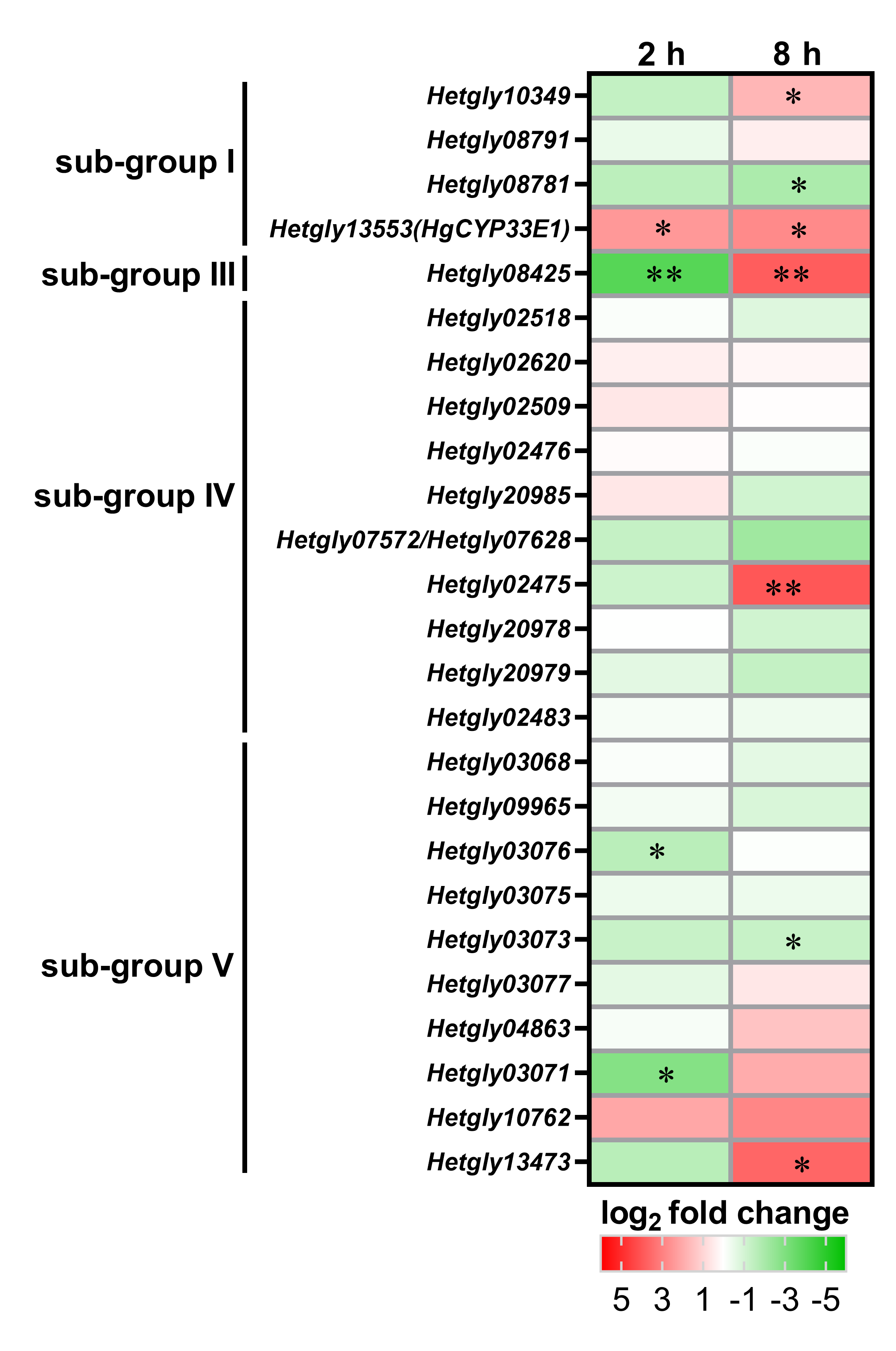


**Supplementary Figure 2.** Heatmap representation for the expression pattern of 26 *HgCYPs* in *H. glycines* post exposure to abamectin treatment. *H. glycines* J2s were exposed to 1 μg/mL abamectin for 2 hours and 8 hours, and qRT-PCR was used to determine *HgCYP* genes expression. Expression levels of genes were shown as the mean of Log_2_ fold change from three biological and three technical replicates. The fold changes of *HgCYPs* expression in treated group were calculated relative to the control (J2s treated with acetone) using the 2^-ΔΔCt^ method. Up-regulated and down-regulated genes are indicated by red and green, respectively; and asterisks in squares indicate statistical differences between the control and the abamectin-treated group (**P < 0.05*, ***P < 0.01*, Student’s t-test).


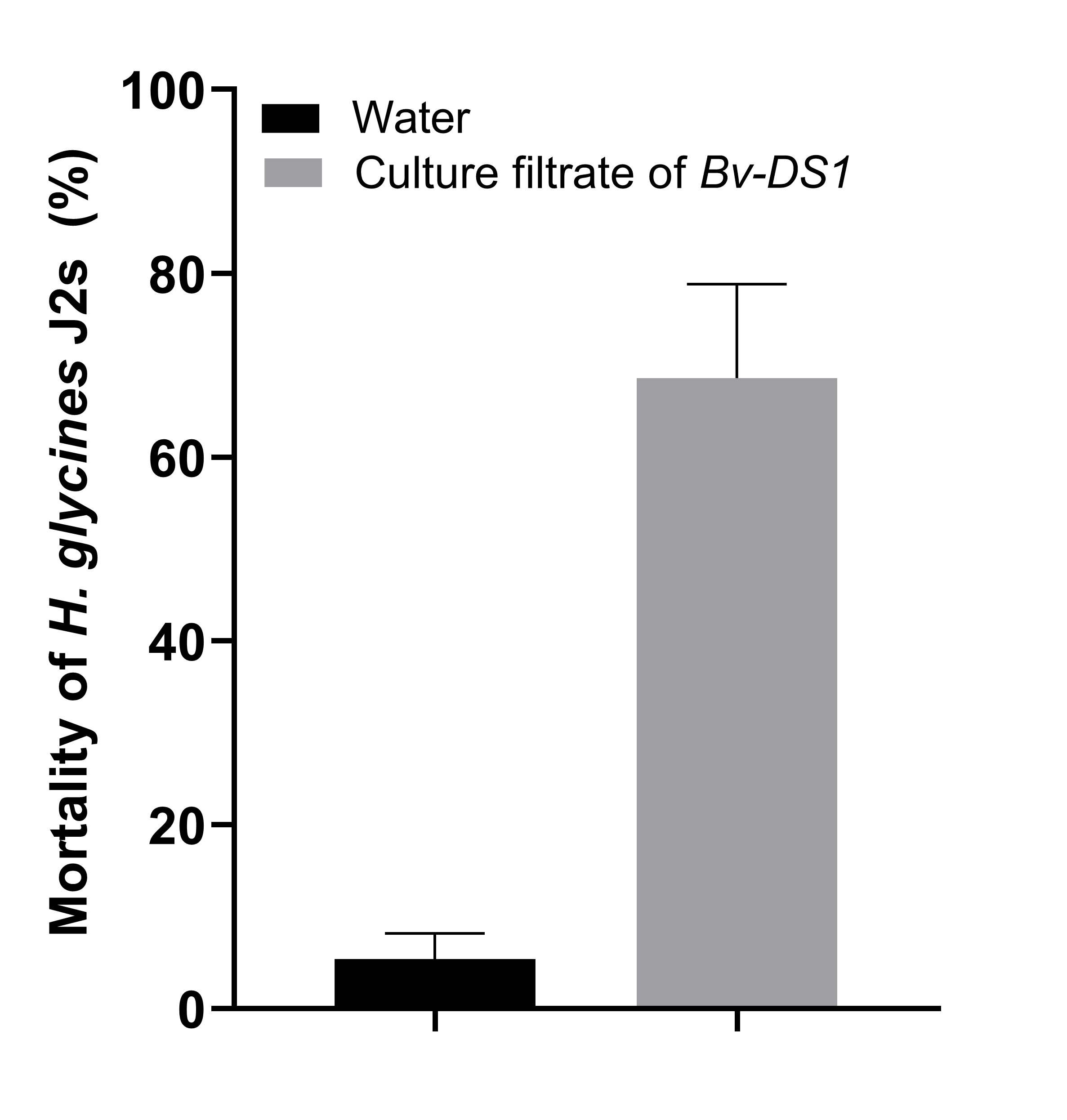


**Supplementary Figure 3.** Filtrate culture of *Bacillus velezensis* YS-AT-DS1 (*Bv-DS1*) affected the mortality of *H. glycines*. Nematode mortality was assessed after a 24-h incubation with the filtrate cultures at room temperature. The data presented represents the mean ± SD of three independent experiments, with eight replicates per treatment.


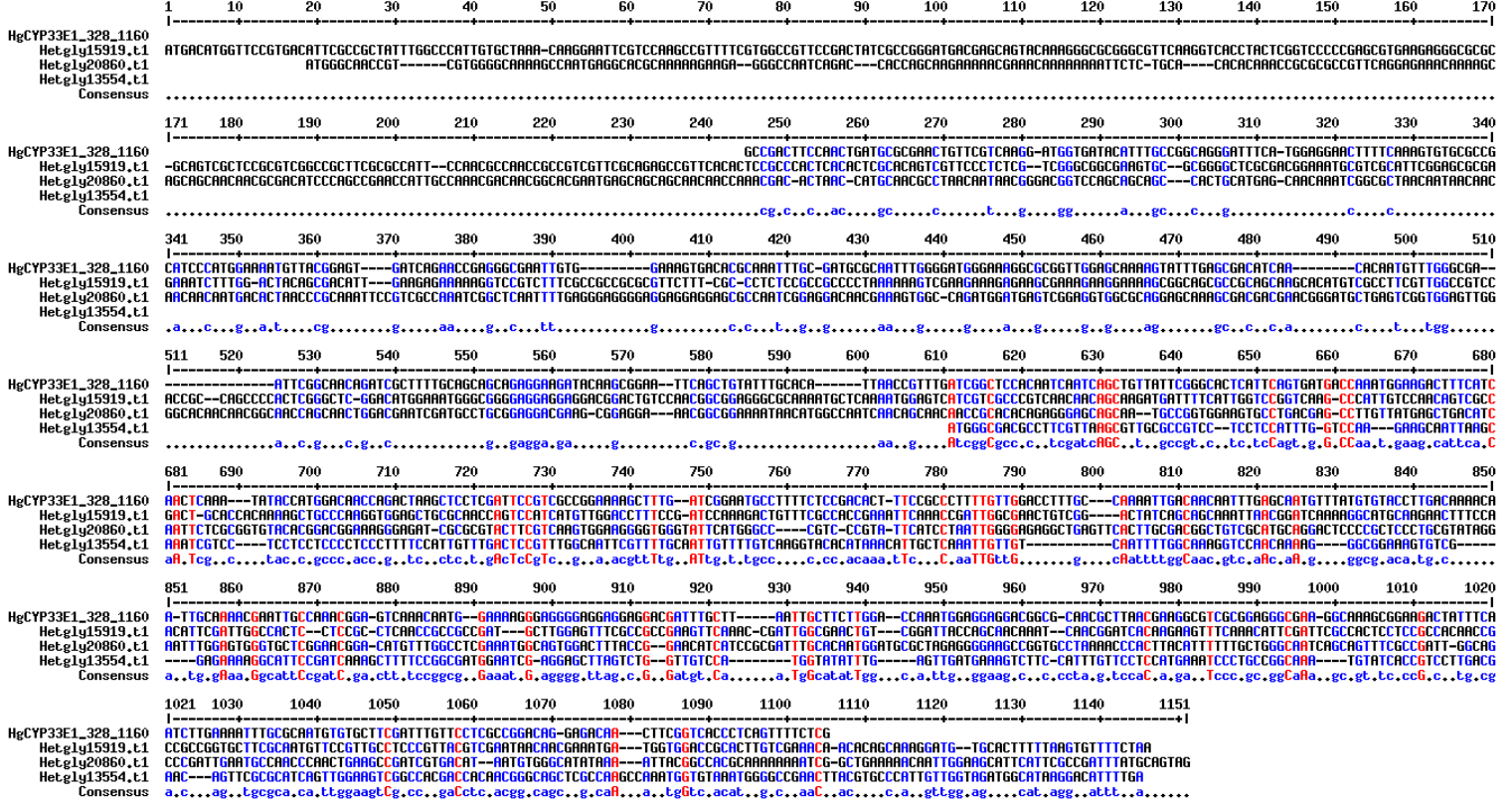


**Supplementary Figure 4.** Analysis of nucleotide sequence similarity between the targeted fragment of *HgCYP33E1* and other three genes of *H. glycines* using MultAlin (https://www.multalin.toulouse.inra.fr/) Bioinformatics Resource Portal.
